# The Mere Substitution Defence of Nudging Works for Neurointerventions Too

**DOI:** 10.1111/japp.12568

**Published:** 2022-02-14

**Authors:** Thomas Douglas

**Affiliations:** ^1^ Oxford Uehiro Centre for Practical Ethics Faculty of Philosophy, University of Oxford Oxford UK; ^2^ Jesus College University of Oxford Oxford UK

## Abstract

Nudges are often defended on the basis that they merely substitute existing influences on choice with other influences that are similar in kind; they introduce no new kind of influence into the choice situation. I motivate the view that, if this defence succeeds in establishing the moral innocuousness of typical nudges, it also establishes the moral innocuousness of an intuitively wrongful neurochemical intervention. I then consider two attempts to rebut this view and argue that both fail. I end by spelling out four stances that the proponent of the defence might adopt in response to my argument.

The staff in a university cafeteria wish to encourage healthier eating choices among customers. It happens that a recently discovered and safe drug is capable of augmenting a person's motivation to eat healthy foods. When inhaled, the drug enters the motivational centres of the brain and chemically induces a mild desire to eat foods with certain flavours – flavours generally possessed by vegetables. Knowing these facts, cafeteria staff arrange to have the drug sprayed into the air via a sprinkler system. Although it is widely known that some cafeterias employ such strategies, and the spray is visible, most customers do not consider the possibility that this particular cafeteria might be employing such measures, and the cafeteria staff do nothing to draw the measure to customers' attention.^1^ The measure results in some customers choosing healthier options than they would otherwise have selected.

Call the intervention described here *Cafeteria Spray*. Many philosophers – especially those operating primarily within a deontological framework – would think that *Cafeteria Spray* wrongs – that is, infringes a *pro tanto* duty owed to – the cafeteria's customers. At least, many would think that this intervention wrongs those among the customers whose choice is influenced by the measure and who have not implicitly consented to it.^2^ Indeed, there is now a burgeoning literature attempting to explain why, precisely, non‐consensual neurochemical interventions, of which *Cafeteria Spray* is an example, wrong their targets. These attempts appeal to the ways in which these interventions interfere with the bodies and minds of their targets, objectify those individuals, or bypass their psychological or rational faculties.^3^

By contrast, many would find it less morally problematic were the cafeteria staff to realise the same outcome through nudging – that is, through (a) altering an individual's environment, (b) in order to influence her choices, (c) by harnessing rapid, low‐effort decision‐making heuristics, such as ‘choose what is most salient’, ‘stick with the default’ or ‘listen to people you recognise’.

Consider this paradigmatic nudge, which I will call *Cafeteria Nudge*:

The staff in a university cafeteria wish to encourage healthier eating choices among the cafeteria's customers. Knowing that foods placed at eye‐level will be more salient to customers than foods placed elsewhere, and that customers will thus be more inclined to choose those foods, they ensure that shelves at eye level are always filled with the healthiest options. Though it is widely known that some cafeterias employ such strategies, most customers do not consider the possibility that this particular cafeteria might be employing them, and the cafeteria staff do nothing to draw the measure to customers' attention. The measure results in some customers choosing healthier options than they would otherwise have selected.^4^

Most would, I venture, find this intervention less morally troubling than *Cafeteria Spray*. Indeed, many commentators hold that nudges are typically morally innocuous, by which I mean that they do not wrong nudgees – even nudgees who are influenced by the nudge and have not implicitly consented to it. Several arguments have been offered in defence of this view.^5^ One of these – and the one that will be my focus – appeals to the claim that nudges typically influence the nudgees' choices only in ways in which their choices would also have been influenced in the absence of the nudge. Typical nudges introduce no new kind of influence; they merely substitute one set of influences for another that is similar in kind.^6^ Discussing nudges that consist in altering the default option faced by the nudgee, one commentator puts it thus:

it's not the case that switching the default option introduces influences into a situation previously devoid of influences; it simply substitutes one set of influences for another. Alterations of the choice architecture thus need not reduce liberty, since the status quo contains patterns of influence that cannot be assumed to be innocent in this regard simply because they emerged in free market interactions.^7^

The argument of interest to me here can be characterised generically as follows:

(1) For some intervention (or set of interventions) *x*, all of the influences that *x* exerts on individuals' choices are influences of a kind that would also have been present in the absence of *x*.

(2) An intervention does not wrong its target(s) if all the influences it exerts on their choices are influences of a kind that would also have applied to those choices in the absence of the intervention.

Therefore

(3) Intervention (or set of interventions) *x* does not wrong its target(s).^8^

I will refer to this argument as ‘the Mere Substitution Defence’ or sometimes simply ‘the Defence’, and to those who invoke it in support of typical nudges as ‘Nudge Defenders’. In this article, I argue that if the Defence succeeds in relation to typical nudges – that is, if it establishes that typical nudges are morally innocuous in the sense specified by (3) – then it also succeeds in relation to an intuitively wrongful neurochemical intervention.

I begin, in the next section, by outlining some difficulties that Nudge Defenders face in adducing the Mere Substitution Defence in support of typical nudges, and indicating how these challenges might be overcome. I then, in the subsequent section, motivate my view that if the Defence succeeds when applied to typical nudges, it also succeeds when applied to an intuitively wrongful neurochemical intervention: namely, *Cafeteria Spray*. Next, I consider two ways in which a Nudge Defender might seek to rebut this view, establishing that the Defence fails in relation to *Cafeteria Spray*, although not in relation to typical nudges. I argue that neither attempt succeeds in showing that the defence fails for all intuitively wrongful variants of *Cafeteria Spray*. Finally, I spell out the options that my argument leaves open to Nudge Defenders, suggesting that each involves a significant concession.

Before proceeding to my argument, however, I must offer an important qualification. The Mere Substitution Defence relies on the thought that influences can be divided into kinds. But tricky questions will arise in individuating kinds of influence. Clearly, all nudges will introduce influences that differ in some minor descriptive ways from the influences that they replace. Consider again *Cafeteria Nudge*. There will presumably be many small differences between the influences introduced by *Cafeteria Nudge* and the influences that they replace. For example, it may be that one staff member, Jack, placed the healthy foods at eye level, whereas, had the nudge not been employed, another staff member, Jill, would have done the food placement. So the nudged customers are influenced by Jack, whereas they would otherwise have been influenced by Jill. Or it may be that the healthy foods were placed at eye level on a Tuesday, whereas the food arrangement would otherwise have been done on a Wednesday. So, the nudged customers are subject to a Tuesday‐originating influence, whereas they would otherwise have been subject to a Wednesday‐originating influence.

If the Mere Substitution Defence is to find any interesting application at all, minor descriptive differences such as these cannot be deemed to be differences in kind. Which differences should be counted as differences in kind, then? In the name of charity to Nudge Defenders, I will assume that only morally relevant differences should be counted, where a morally relevant difference is one that renders the substituting influence more morally problematic than the substituted influence. Differences that are not morally relevant need not trouble the proponent of the Defence since one cannot wrong someone by replacing one influence with another that is no more problematic. Or so I will assume. I will henceforth take it as read that differences in ‘kind’, for the purposes of the Mere Substitution Defence, must be morally relevant differences.

## 1 The Mere Substitution Defence Applied to Nudging

Nudge Defenders adduce the Mere Substitution Defence in support of typical nudges. That is to say, they maintain that, when ‘intervention (or set of interventions) *x*’ is taken to stand for ‘typical nudges’ the Mere Substitution Defence is a valid argument with true premises. But is it? In this section I outline some challenges for the view that it is, before explaining how Nudge Defenders might respond to these challenges. Throughout, I use one typical nudge *– Cafeteria Nudge –* as an exemplar.

Recall that *Cafeteria Nudge* involves arranging various meal offerings such that healthier foods lie at eye level, while less healthy foods are placed elsewhere. By increasing the salience of the healthy foods, it succeeds in influencing some customers to choose these foods when they would not otherwise have done so.

Does this intervention introduce a new kind of influence into the choice situation? Nudge Defenders will hold that it does not. They may note that some foods will need to be placed at eye level and some in other locations. Thus, some foods will be more salient than others. The nudge merely arranges things such that this salience militates in favour of choosing the healthy option.

However, as many protagonists in the philosophical debate on nudging have noticed, there is at least one significant *–* and possibly morally relevant *–* difference between the influences created by most nudges and the influences they replace. Whereas, the influences introduced by nudges, including *Cafeteria Nudge*, are created by others with the intention of influencing the choices or conduct of nudgees, the influences they replace are in many cases ‘accidental’: they are either not the product of intentional action or not the product of an intention to influence choices.^9^

Why think that this difference could be morally relevant? Perhaps because it could have an impact on autonomy. Some would argue that one is sometimes less autonomous when one is intentionally influenced by others than when one is influenced by accidental features of one's situation, even if the influences are otherwise similar. This view is implied, for instance, by conceptions of autonomy according to which autonomy requires independence from the control, coercion, or manipulation of others.^10^ It is also able to accommodate some widely held intuitions. As J.S. Blumenthal‐Barby notes in discussing the distinction between accidental and intentional influences, ‘[t]o see the importance [of this distinction] we need only consider why it is that we think that something like slavery is so morally egregious: it is not just because the slave is not able to govern himself, it is because he is governed by someone else. The master has imposed his will on the slave in a way that the slave would not endorse.’^11^ It could be argued, then, that nudgers wrong nudgees because, in replacing accidental with intentional influences, the nudger brings it about that he controls, manipulates or imposes his will on the nudgee. In this vein, Till Grüne‐Yanoff suggests that nudge policies ‘are manipulative [in part] because the government employs them with the intention of affecting people's choices’,^12^ while Daniel Hausman and Brynn Welch maintain that ‘[t]here remains an important difference between choices that are intentionally shaped and choices that are not. Even when unshaped choices would have been just as strongly influenced by deliberative flaws, calculated shaping of choices still imposes the will of one agent on another.’^13^

There is also another important *–* and possibly morally relevant *–* way in which the influences introduced by nudges often differ from those they replace: they are likely to differ in direction.^14^ That is, they are likely to militate in favour of different choices. Proponents of the Mere Substitution Defence often note that the direction of influences produced by a nudger could have come about accidentally. For example, the healthy foods could have been placed at eye level even if the foods were arranged randomly, and if they were, the customers would have been influenced in precisely the same direction as they in fact were by the nudge. However, although this could have been the case, it will often not be true that it would have been the case. Instead, in many cases, the substituting influences will militate in favour of different choices than the substituted influences. In the absence of *Cafeteria Nudge*, it is likely that the food arrangement would have been different and would have militated in favour of choosing different foods. Indeed, if there were not at least a chance that the nudge would alter the direction of influence, there would have been no point in pursuing it.

As with the difference between accidental and intentional influences, it is possible that differences in direction of influence are, at least in some cases, morally relevant. Suppose an individual customer of our imagined cafeteria weakly but autonomously prefers to eat only unhealthy foods at the point at which he enters the cafeteria, and, as it happens, in the absence of *Cafeteria Nudge*, product placement would have militated in favour of his choosing unhealthy foods. Against this background, the influence introduced by *Cafeteria Nudge*, which favours the selection of healthy foods, works against this customer's autonomous preference, whereas the influence it replaces would have been aligned with the preference.^15^ Although this will of course depend on precisely how we conceive of autonomy, it might follow that *Cafeteria Nudge* has compromised the autonomy of, and perhaps thereby wronged, this customer.

How might Nudge Defenders respond to these challenges? One option would be to severely restrict the scope of the Defence so that it applies only to cases in which, had the intervention not been employed, the targeted individuals would nevertheless have been subjected to intentional influences with the same direction. However, this would be a major concession; it would take almost all nudges beyond the scope of the Defence. More likely, then, Nudge Defenders will respond by denying that differences in intentionality and direction are always morally relevant and thus that they always block the application of the Mere Substitution Defence to typical nudges. Typical nudges can alter the intentionality and direction of the influences exerted on nudgees without resulting in any change in the ‘kind' of influences brought to bear on nudgees.

## 2 The Mere Substitution Defence Applied to *Cafeteria Spray*

We will return to this idea below. But for now, let us turn to consider the neurochemical intervention, *Cafeteria Spray*. Recall that *Cafeteria Spray* involves dispersing a drug that, when inhaled, chemically induces a mild desire to eat foods with certain flavours *–* flavours generally possessed by vegetables. Can the Mere Substitution Defence be adduced in support of this intervention?

It might initially seem obvious that it cannot. It might seem that, in spraying the chemical into the air, cafeteria staff introduce a new kind of influence *–* an influence that differs in kind from the influences that would otherwise have been present.^16^ Initial appearances can be deceptive, however. In this section, I assume that the Mere Substitution Defence establishes the moral innocuousness of typical nudges, and I explain why I think that, on this assumption, the Defence also establishes the moral innocuousness of *Cafeteria Spray*. To be clear, my argument in this section should be understood only as an attempt to *motivate* this view, not as a full defence of it; in the next section, I will buttress the view by outlining and rebutting two attempts to refute it.

My argument begins with the observation that all of our choices are influenced by certain pre‐existing motivational states and dispositions (henceforth simply ‘motivations’). Suppose that you are deciding between buying a bottle of water and a cup of coffee. You opt for the coffee. Clearly, in making this choice, you will be influenced by a range of pre‐existing motivations such as your level of thirst, your desire to experience the taste of coffee and water, whether you plan to engage in activities that you think could be aided by a caffeine boost, and so on. Let us refer to these pre‐existing motivations collectively as the motivational context of your choice.

This motivational context will be at least influenced (and perhaps determined) by chemical features of your brain, body, and environment *–* by the chemical context of your choice. For instance, your level of thirst is influenced by the level of a hormone *–* angiotensin II *–* in the ventricles of your brain, which is in turn influenced by your blood pressure and the concentration of your bodily fluids, which are in turn influenced by the salt content of your food. More generally, the chemistry of our brains, bodies, and environment influences the motivational context of our choices, and thus the choices themselves.

The influence of chemistry on our motivations, and thus choices, is clearest in cases where an unusual chemical state leads to unusual motivations, as, for example, where the hormones of early pregnancy lead to cravings for certain foods, very low blood sugar levels produce extreme hunger and impatience, or the administration of certain brain‐active drugs induces apathy. But work in psychology and neuroscience has also revealed numerous less obvious chemical influences on our motivations and thus choices. For example, research in empirical moral psychology has identified a role for the serotonergic neurotransmitter system in modulating motivations and decisions regarding whether to cooperate,^17^ how to respond to unfair treatment,^18^ and which option to take when presented with a putative moral dilemma.^19^ Of course, from a philosophical standpoint, it should not be surprising that chemical factors affect our motivations; many accept that the mind supervenes on the physico‐chemical properties of our brains, and even mind–body dualists, who reject this view, typically concede that bodily states can exert a causal influence on the mind, and thus on our choices.^20^

I take it to be clear, then, that our motivations, and thus choices, are influenced by chemical factors. In some rare cases, these chemical factors are deliberately controlled, either by ourselves or by others, for the purposes of influencing our motivation and thus choices. But for the most part, the chemical factors that bear on our motivations and choices are a matter of accident *–* they are determined either by genetic factors or by features of our environment that were not created with the intention of influencing our choices. Our choices are influenced by accidental chemical features of the world.

With these thoughts in mind, let us return to consider *Cafeteria Spray*. This intervention exerts an influence on the choices of customers exposed to it, and it does so through chemical means. However, in light of what has just been said, those customers' choices would in any case have been subject to accidental chemical influences. The actions of the cafeteria staff simply replace one set of chemical influences with another. Of course, those new influences were intentional, whereas the chemical influences they replace were likely accidental. Moreover, they likely differ in direction. However, as we have seen, the analogous claims are true of many typical nudges also. To adduce the Mere Substitution Defence in support of typical nudges, Nudge Defenders must allow that the differences in intentionality and direction are not *–* or not always *–* morally relevant differences. Only then will they be entitled to claim that, although typical nudges often replace accidental with intentional influences on choice, they nevertheless introduce no new kind of influence, so the Mere Substitution Defence still applies. But on that assumption, it is not obvious why we should think that differences in intentionality and direction *are* morally relevant with respect to *Cafeteria Spray*. And if they are not, the Defence can, it seems, be adduced in support of that intervention also.

## 3 Responses

An obvious objection to my argument of the previous section would hold that it relies on an overly coarse‐grained account of the kind of intervention exemplified by *Cafeteria Spray*. I characterised that kind of intervention as a chemical influence on motivation and thus choice. But we could of course characterise it more narrowly. For example, we could characterise the kind of influence exemplified by *Cafeteria Spray* as the influence on motivation and thus choice of chemicals contained in airborne particles. Perhaps, had the cafeteria staff not released the spray, the cafeteria air would have contained no airborne particles of a kind that influence motivation. On this finer‐grained specification of the kind of influence involved here, we can say that, had the cafeteria staff not pursued *Cafeteria Spray*, no influence of the same kind would have existed.^21^

But recall that, to qualify as a difference in kind, the substituting and substituted influences must differ in a morally relevant way. Yet the present objection appears to invoke a morally irrelevant difference; it is not clear that or why it is more morally problematic to have one's motivations influenced by chemicals contained in airborne particles than to have them influenced by non‐airborne but otherwise similar chemical influences, such as chemicals on surfaces that are absorbed through the skin or chemicals in foods that we eat.^22^

Our question, then, should be this: are there morally relevant differences between the influences introduced by *Cafeteria Spray* and those they replace? In what follows, I consider two attempts to show that there are, and thus to block the application of the Mere Substitution Defence to *Cafeteria Spray*.

### 3.1 Differences in Strength and Phenomenal Character

The first attempt appeals to the strength and phenomenal character of the influences introduced by *Cafeteria Spray*. It is natural to think that the influences introduced by *Cafeteria Spray* would be rather strong, and certainly stronger than the influences they replace. It is also plausible that they would differ in their phenomenal character *–* in how they feel to the customers. It may be, for example, that the motivations produced by the drug feel irresistible in a way that the motivations produced by the background chemical milieu do not. Perhaps these differences are morally relevant and qualify as differences in kind.

This suggestion faces at least two problems, however. First, it would, of course, be possible to more finely specify *Cafeteria Spray* such that the influences it introduces are neither stronger than, nor different in phenomenal character to, those they replace. There would then be no strength‐based or phenomenal difference in kind between the substituted and substituting influences. The Mere Substitution Defence could then be adduced in support of this more fully specified variant of *Cafeteria Spray*, yielding the counter‐intuitive result that it is not wrongful.

Of course, it might be claimed that this is of no practical import: in almost all neurochemical interventions that might in fact be employed, there would be at least minor differences in phenomenal character and strength between the influences introduced by the intervention and those they replace. The chance that there is no difference is so small that, for practical purposes, it can be ignored. And minor differences in strength and phenomenal character are, it might be claimed, sufficient to block the Mere Substitution Defence.

However *–* and this brings us to the second problem *–* Nudge Defenders must allow that minor differences in strength or phenomenal character are morally irrelevant. If they do not, they will undermine their own defence of nudging since, although this has not been widely acknowledged, many typical nudges will also create influences that are somewhat stronger than or different in phenomenal character to those they replace. Consider again *Cafeteria Nudge*. Suppose that, had this nudge not been employed, foods would have been distributed not in such a way as to produce healthy choices, but simply so as to minimise re‐stocking time. This, let us suppose, would have resulted in bottles of water being placed at eye level. Would this have resulted in an influence identical in strength and phenomenal character to that actually exerted by the nudge *–* that is, the placement of healthy foods at eye level? Probably not. Most likely, the salience of bottled water placed at eye level will somewhat differ, in both its phenomenal character and motivational strength, from the salience of healthy foods placed in the same position.

Given possibilities such as this, Nudge Defenders must hold that, at least within a certain range, differences in strength and phenomenal character are not morally relevant. But on that view, if *Cafeteria Spray* produced influences that differ only slightly in strength and phenomenal character from those they replace, which it clearly might, then the differences will be morally irrelevant, and the Mere Substitution Defence can still apply.

### 3.2 Intentional Bodily Influence

A second attempt to block the application of the Mere Substitution Defence to *Cafeteria Spray* would appeal to the role of the body in mediating the influence exerted by this intervention. The influences introduced by *Cafeteria Spray* were produced via intentionally and directly (that is, without psychological mediation) influencing bodily states. The cafeteria staff intentionally alter the customers' neurochemical, and thus bodily, states. Moreover, they alter them not by engaging psychological processes, such as perception, but through brute physico‐chemical mechanisms *–* mechanisms with no psychological correlates. By contrast, the influences that are replaced by this intervention *–* the background chemical influences on motivation *–* were not, we may assume, produced via intentional and direct bodily influence.

This difference may be morally relevant since it may affect whether the right to bodily integrity is implicated. A right to bodily integrity, understood here as a right against bodily interference,^23^ is widely accepted in moral, legal, and political philosophy, where it has played an especially prominent role in discussions of the wrongfulness of non‐consensual sex, organ harvesting, and medical intervention.^24^ Intentionally and directly influencing a person's internal bodily chemistry, at least when done without that person's consent, is often thought to infringe a person's right to bodily integrity. So the influences introduced by *Cafeteria Spray* might be thought to infringe this right. On the other hand, merely allowing accidental features of a person's bodily and environmental chemical milieu to exert an influence would not standardly be taken to infringe this right. This suggests that the background influences replaced by *Cafeteria Spray* would not infringe this right. *Cafeteria Spray* may, then, infringe the customers' rights, and in a way that they would not, in the absence of the nudge, have been infringed; it may substitute bodily‐integrity‐infringing for non‐bodily‐integrity‐infringing forms of influence.

Perhaps here we have found a way of blocking the application of the Mere Substitution Defence to *Cafeteria Spray*. If we have, we will also, I think, have found a way of morally separating *Cafeteria Spray* from typical nudges, for typical nudges surely do not infringe the right to bodily integrity. Of course, insofar as nudges affect the mind, and the mind supervenes on the brain, nudges affect neural, and thus bodily, states. But it is doubtful that they are intended to do so, and it is doubtful that the right to bodily integrity protects against unintended bodily influence. Furthermore, the bodily alterations produced by nudging are produced by engaging psychological processes, whereas the right to bodily integrity is normally understood as protecting only against direct influence on the body. This gives us a further reason to deny that nudges infringe the right to bodily integrity.

Appealing to the right to bodily integrity to explain the wrongfulness of *Cafeteria Spray* is, however, a risky strategy for the Nudge Defender to take, for although nudges do not infringe the right to bodily integrity as it is normally understood, they plausibly do infringe the deeper rights which ground bodily integrity.

Let me explain. The right to bodily integrity is seldom taken to be a rock‐bottom feature of morality. Rather, it is normally thought to derive from some deeper right(s), such as rights of personal sovereignty or personal autonomy (henceforth, ‘rights over the person’).^25^ On this view, to infringe the right to bodily integrity is just to infringe rights over the person, and to do so in a particular kind of way *–* by interfering with the person's body. However, rights over the person can also be infringed by influences that do not interfere with the body. And indeed, they are precisely the rights that many opponents of nudging would claim are threatened by nudges by virtue of the way in which nudges interfere with our motivations and choices. Those opponents claim that, in replacing accidental with intentional influences on motivation and choice, nudges replace influences that do not infringe rights over the person with influences that *–* perhaps because they are manipulative or controlling *–* do.

Of course, Nudge Defenders can deny this. They can deny that intentional influences on motivation always infringe rights over the person. But can they do so in a way that is consistent with holding that the bodily influence introduced in *Cafeteria Spray does* infringe such rights?

Those who defend nudging against the objection that it infringes rights of personal sovereignty or personal autonomy normally do so by arguing that, within certain constraints *–* which nudges typically satisfy *–* influencing another's motivations does not threaten such autonomy or sovereignty *–* at least, not to the degree that it infringes any right. The most frequently mentioned constraints are that the influence must be type transparent (i.e. people must be made aware of the fact that influences of this type are sometimes employed),^26^ that it must be easy to avoid,^27^ and that it must not harness any irrationality in the influencee.^28^ However, if influences on motivation infringe no rights over the person when they meet these criteria, we might suspect that the same will be true of influences on the body when they meet analogous criteria. Moreover, it is plausible that the bodily influences introduced in *Cafeteria Spray* could meet these criteria. There seems no reason to suppose that *Cafeteria Spray* harnesses any irrationality.^29^ And if we suppose that the government informs citizens that some cafeterias are using this spray, and that it is easy for customers to avoid such cafeterias *–* for example, by asking staff whether they are employing the spray, and dining elsewhere if they are *–* then the type transparency and easy avoidability conditions will also be met. Nevertheless, it is intuitively plausible that this version of *Cafeteria Spray* remains wrongful.

The challenge for proponents of the Mere Substitution Defence is, then, to explain why the intentional, direct influence on the body introduced in *Cafeteria Spray* infringes rights over the person, even when it is type transparent, is easy to avoid, and harnesses no irrationality, yet the intentional influences on motivation and choice introduced by typical nudges do not infringe those rights. Perhaps proponents of the Mere Substitution Defence will be able to answer this challenge. This is, however, work that remains to be done.

## 4 Implications

I have now motivated the view that, if the Mere Substitution Defence succeeds in relation to typical nudges, then it also succeeds in relation to *Cafeteria Spray*. I have also considered two attempts to refute that view. I argued that these attempts fail to block the application of the Defence to some intuitively wrongful variants of *Cafeteria Spray –* namely, those in which the chemical influences introduced by the spray differ only slightly in strength and phenomenal character from background chemical influences, and in which the intervention is type transparent, is easy to avoid, and harnesses no irrationality.

How might a Nudge Defender respond to my argument? There are four broad routes available.

One option would be to bite the bullet *–* to accept that the relevant variants of *Cafeteria Spray* are morally innocuous: they do not wrong those whom they target, counter‐intuitive as this may be.

Another option would be to concede that some typical nudges do wrong those subjected to them *–* to concede, in other words, that the Mere Substitution Defence fails in its original application. For example, one could allow that typical nudges wrong those subjected to them whenever they replace accidental with intentional influences.

I take it that the Nudge Defender will find neither of these options attractive; she will want to sustain the view that typical nudges are morally innocuous without being committed to the moral innocuousness of any intuitively wrongful variants of *Cafeteria Spray*. This brings us to the third option: the Nudge Defender might confront my argument head‐on. She might seek to establish, contrary to my argument in the previous section, that, whereas typical nudges fall within the scope of the Mere Substitution Defence, intuitively wrongful variants of *Cafeteria Spray* do not.

The most promising variant of this strategy would, I think, develop the line that we were pursuing at the end of the previous section. The Nudge Defender might seek to show that intentional and direct influences on the body infringe rights over the person in a way that intentional influences on motivation do not. However, considerable further philosophical work would need to be done to establish this result, and I see no reason to assume in advance that this project will succeed.

Finally, the fourth option would be for the Nudge Defender to simply insist *–* without arguing *–* that the influences introduced by *Cafeteria Spray* differ in kind from those that they replace, whereas those introduced by typical nudges do not. In defence of this view, she might note that it aligns well with widely shared intuitions. Many people will, I suppose, intuit that *Cafeteria Nudge* merely substitutes influences but *Cafeteria Spray* does not. This, the Nudge Defender might claim, puts the burden of proof on anyone who wishes to deny that there is a difference between these interventions with respect to the Mere Substitution Defence.

However, this response also comes at a significant cost. We do not, in philosophy, typically take bare intuition as a fully satisfactory basis for accepting a view. We have a stronger basis for accepting an intuitively plausible view when we can also offer a philosophical rationale for the view, and a weaker basis when we lack one. Taking this fourth response, then, leaves the Mere Substitution Defence of nudging unsatisfyingly exposed.

## NOTES

1 I make the stipulations contained in this sentence and the previous two in order to ensure that the intervention described in this case is analogous to typical nudges, with which I will compare it below, in three important ways: it is ‘type transparent’ (infra, note 26) and avoidable, though not clearly consensual.

2 On some accounts of rights, to wrong a person – viz., to infringe a *pro tanto* duty owed to her – is to infringe her rights, but I will not commit myself to such accounts. Nevertheless, I will assume that infringing someone's rights is *one way* of wronging her. I use the term ‘wrongful’ to refer to actions that wrong someone. Thus, on my usage, wrongfulness is different from wrongness. Wrongness implies all‐things‐considered impermissibility, whereas wrongfulness does not.

3 See, for example, Bublitz, Jan Christoph, and Reinhard Merkel. 2014. “Crimes Against Minds: On Mental Manipulations, Harms, and a Human Right to Mental Self‐Determination.” *Criminal Law and Philosophy* 8(1): 51–77; Bublitz, Jan Christoph. 2016. “Moral Enhancement and Mental Freedom.” *Journal of Applied Philosophy* 33(1): 88–106; Shaw, Elizabeth. 2014. “Direct Brain Interventions and Responsibility Enhancement.” *Criminal Law and Philosophy* 8(1): 1–20; Shaw, Elizabeth. 2015. “The Use of Brain Interventions in Offender Rehabilitation Programs: Should It Be Mandatory, Voluntary, or Prohibited?” In *Handbook of Neuroethics*, edited by Jens Clausen and Neil Levy, 1381–98. Dordrecht: Springer; Bennett, Christopher. 2018. “Intrusive Intervention and Opacity Respect.’ In *Treatment for Crime*, edited by David Birks and Thomas Douglas, 256–74. Oxford: Oxford University Press. These works all consider the use of non‐consensual neurochemical interventions for the purposes of crime prevention or criminal rehabilitation and within the context of criminal justice. However, if non‐consensual neurochemical interventions are wrongful in criminal justice contexts, in which those subject to them might be thought to have made themselves liable to certain intrusive interventions, it is very plausible that they would be wrongful also in many other contexts. And indeed objections have also been discussed – although somewhat less fully – to non‐consensual neurochemical interventions employed for other purposes and in other contexts, including to treat psychiatric disorders and manipulate the outcomes of elections. See, for example, Sjöstrand, M., and G. Helgesson. 2008. “Coercive Treatment and Autonomy in Psychiatry.” *Bioethics* 22(2): 113–20; Paulo, Norbert, and Christoph Bublitz. 2019. “Pow(d)er to the People? Voter Manipulation, Legitimacy, and the Relevance of Moral Psychology for Democratic Theory.” *Neuroethics* 12(1): 55–71.

4 This case is modified from Thaler, Richard H., and Cass R. Sunstein. 2008. *Nudge: Improving Decisions about Health, Wealth, and Happiness*. New Haven, CT: Yale University Press, pp. 1–2.

5 Wilkinson, T. Martin. 2013. “Nudging and Manipulation.” *Political Studies* 61(2): 341–55; Hausman, Daniel M., and Brynn Welch. 2010. “Debate: To Nudge or Not to Nudge.” *Journal of Political Philosophy* 18(1): 123–36; Thaler, Richard H., and Cass R. Sunstein. 2003. “Libertarian Paternalism.” *American Economic Review* 93(2): 175–79; Anderson, Joel. 2010. “Nudge: Improving Decisions about Health, Wealth, and Happiness, Richard H. Thaler and Cass R. Sunstein (Yale University Press, 2008), pp. x+293 pages. [Paperback edition, Penguin, 2009, 320 pages.].” *Economics and Philosophy* 26(3): 369–76.

6 Thaler and Sunstein 2003 op. cit., p. 175; Thaler and Sunstein 2008 op. cit., pp. 2–4, p. 243, p. 247; Anderson op. cit., pp. 372–73; Hausman and Welch op. cit. note 5, pp. 132–33. Note that, as I understand it, it does not follow from the claim that nudges introduce no new kind of influence that they introduce no new object or feature into the nudgee's environment. Suppose that supermarket staff place colourful nutritional labels on food shelves where previously there were no labels. The staff have introduced new objects and features into the customers' environment. Nevertheless, I take it to be possible that no new kind of influence is produced. It may be that the impact of the nutritional labels on customers' choices merely replaces a similar impact that some other existing feature of their environment – perhaps the food packaging itself – would otherwise have had.

7 Anderson op. cit.

8 The conclusion of this argument is not normally explicitly stated. Some have seemed prepared to conclude, partly on the basis of (1), that the specified interventions are entirely morally unproblematic or ethically ‘neutral’. See, for example, Kalle Grill's interpretation of Thaler and Sunstein's version of the argument, in Grill, Kalle. 2014. “Expanding the Nudge: Designing Choice Contexts and Choice Contents.” *Rationality, Markets and Morals* 5(90): 139–62, pp. 142–43. I will, conservatively, attribute to proponents of the argument only the weaker conclusion that the interventions are in one particularly important respect unproblematic: they do not wrong those whom they target (that is, they are not, in my sense of the term, wrongful).

9 See, for example, Hausman and Welch op. cit., esp. p. 133; Grüne‐Yanoff, Till. 2012. “Old Wine in New Casks: Libertarian Paternalism Still Violates Liberal Principles.” *Social Choice and Welfare* 38(4): 635–45; Blumenthal‐Barby, J. S. 2014. “A Framework for Assessing the Moral Status of “Manipulation”.” In *Manipulation: Theory and Practice*, edited by C. Coons and M. Weber, 121–34. New York: Oxford University Press, pp. 125–26; Kipper, Jens. 2020. “Irresistible Nudges, Inevitable Nudges, and the Freedom to Choose.” *Moral Philosophy and Politics* 8(2): 11–9. https://doi.org/10.1515/mopp-2020-0013, see esp. pp. 11–2.

10 Clayton, Matthew. 2006. *Justice and Legitimacy in Upbringing*. Oxford: Oxford University Press, p. 104; Clayton, Matthew. 2002. “Individual Autonomy and Genetic Choice.” In *A Companion to Genethics*, edited by Justine Burley and John Harris, 191–205. Malden, MA: Blackwell, p. 201; Raz, Joseph. 1986. *The Morality of Freedom*. Oxford: Clarendon Press, pp. 377–78; Manson, Neil C., and Onora O'Neill. 2007. *Rethinking Informed Consent in Bioethics*. Cambridge: Cambridge University Press, p. 18. On an alternative view, what matters is not whether one is subjected to controlling influence by others, but whether one is influenced in a way that one resisted or would have resisted had one noticed it. See, for the classic statement of this view, Christman, John. 1991. “Autonomy and Personal History.” *Canadian Journal of Philosophy* 21(1): 1–24, p. 11. I do not discuss this view further, but I hope it will be clear that my arguments below apply equally to it.

11 Blumenthal‐Barby op. cit., p. 126.

12 Grüne‐Yanoff op. cit., p. 636.

13 Hausman and Welch op. cit., note 9, p. 133.

14 This difference has been less frequently noted in the literature on nudging than the difference between accidental and intentional influences, perhaps because it is generally assumed that the direction of influence in nudging is unproblematic. But see, for an article that does acknowledge this difference, and its potential moral relevance, Barton, Adrien, and Till Grüne‐Yanoff. 2015. “From Libertarian Paternalism to Nudging – and Beyond.” *Review of Philosophy and Psychology* 6(3): 341–59, p. 348.

15 On some conceptions of nudging, nudges necessarily militate in favour of choices that conform to the agent's own reasons. The intervention I describe here may fail to qualify as a nudge, on this conception, although this will depend on how, exactly, we understand reasons. For example, if reasons are understood as objective normative considerations – normative considerations that are constitutively independent of a person's contingent preferences – then this intervention may militate in favour of conformity to reasons even though it acts against the nudgee's prior preferences.

16 Admittedly, it could be objected that, had the cafeteria staff not released the spray, they would have been influencing the customers through maintaining the absence of the spray and thus, relatively speaking, favouring less healthy food choices. That influence, it might further be maintained, is of the same kind as the influence introduced by *Cafeteria Spray*. Note, however, that if this objection is generalised, so as to hold that omitting to introduce intervention *x* constitutes an influence of the same kind as introducing intervention *x*, then the Mere Substitution Defence will, implausibly, apply to *any* intervention that influences a person's choices.

17 Knutson, Brian, Owen M. Wolkowitz, Steve W. Cole, Theresa Chan, Elizabeth A. Moore, Ronald C. Johnson, Jan Terpstra, *et al*. 1998. “Selective Alteration of Personality and Social Behavior by Serotonergic Intervention.” *American Journal of Psychiatry* 155(3): 373–79; Tse, Wai S., and Alyson J. Bond. 2002. “Serotonergic Intervention Affects Both Social Dominance and Affiliative Behavior.” *Psychopharmacology* 161(3): 324–30; Tse, Wai S., and Alyson J. Bond. 2003. “Reboxetine Promotes Social Bonding in Healthy Volunteers.” *Journal of Psychopharmacology* 17(2): 189–95; Wood, Richard M., James K. Rilling, Alan G. Sanfey, Zubin Bhagwagar, and Robert D. Rogers. 2006. “Effects of Tryptophan Depletion on the Performance of an Iterated Prisoner's Dilemma Game in Healthy Adults.” *Neuropsychopharmacology* 31(5): 1075–84.

18 Crockett, Molly J., Luke Clark, Golnaz Tabibnia, Matthew D. Lieberman, and Trevor W. Robbins. 2008. “Serotonin Modulates Behavioral Reactions to Unfairness.” *Science* 320(5884): 1739; Crockett, Molly J., Annemieke Apergis‐Schoute, Benedikt Herrmann, Matthew D. Liberman, Ulrich Müller, Trevor W. Robbins, and Luke Clark. 2013. “Serotonin Modulates Striatal Responses to Fairness and Retaliation in Humans.” *Journal of Neuroscience* 33(8): 3505–13.

19 Crockett, Molly J., Luke Clark, Marc D. Hauser, and Trevor W. Robbins. 2010. “Serotonin Selectively Influences Moral Judgment and Behavior Through Effects on Harm Aversion.” *Proceedings of the National Academy of Sciences of the United States of America* 107(40): 17433–38.

20 Leibniz famously subscribed to a view which denies this: mind–body parallelism. He held that, although mental and physical events seem to cause one another, this is only because God has pre‐established a harmony between them: in fact, all mental events have only mental causes, and bodily events have only bodily causes. (See von Leibniz, Gottfried Wilhelm. 1989. *Philosophical Essays*. Edited and translated by Roger Ariew and Daniel Garber. Indianapolis, IN: Hackett, esp. p. 223) I cannot go into the arguments for and against mind–body parallelism here. I hope it suffices merely to note that it is not a popular philosophical position. Mind–body dualism – according to which the mind and body are separate substances – is somewhat more popular. However, mind–body dualism is consistent with the view that mental events have bodily causes, and most adherents to it have accepted as much. One prominent variety of dualism allows that mental and bodily events may cause one another (interactionism), while another (epiphenomenalism) holds that mental events are caused by physical events but not the reverse.

21 Interestingly, it is not obvious that this objection is empirically warranted. There is growing evidence that common airborne pollutants can influence brain structure and function, in some cases with measurable psychological effects. See Chen, Jiu‐Chiuan, Xinhui Wang, Gregory A. Wellenius, Marc L. Serre, Ira Driscoll, Ramon Casanova, John J. McArdle, *et al*. 2015. “Ambient Air Pollution and Neurotoxicity on Brain Structure: Evidence from Women's Health Initiative Memory Study.” *Annals of Neurology* 78(3): 466–76; Calderón‐Garcidueñas, Lilian, Aristo Vojdani, Eleonore Blaurock‐Busch, Yvette Busch, Albrecht Friedle, Maricela Franco‐Lira, Partha Sarathi‐Mukherjee, *et al*. 2015. “Air Pollution and Children: Neural and Tight Junction Antibodies and Combustion Metals, the Role of Barrier Breakdown and Brain Immunity in Neurodegeneration.” *Journal of Alzheimer's Disease* 43(3): 1039–58; Schikowski, Tamara, Mohammad Vossoughi, Andrea Vierkötter, Thomas Schulte, Tom Teichert, Dorothee Sugiri, Karin Fehsel, *et al*. 2015. “Association of Air Pollution with Cognitive Functions and Its Modification by APOE Gene Variants in Elderly Women.” *Environmental Research* 142: 10–6.

22 There may be cases in which this distinction is morally relevant in a derivative sense. Suppose *A* has promised *B* not to influence her through the release of airborne particles; in this context, the normative status of an influence applied by *A* on *B* may clearly depend on whether the influence involves the release of airborne particles. However, in this case the moral relevance of the distinction derives from the more fundamental moral relevance of the distinction between promise‐violating and promise‐respecting forms of influence. My claim is merely that the distinction between chemical influence via airborne droplets and other forms of chemical influence is obviously not in itself – that is, non‐derivatively – morally relevant.

23 Other terms have also been used to refer to what I am calling the right to bodily integrity. For instance, it is sometimes referred to as a right against bodily ‘trespass’, especially when it is taken to derive from rights of self‐ownership or personal sovereignty. See Thomson, Judith Jarvis. 1990. “Trespass and First Property.” In her *The Realm of Rights*, 205–26. Cambridge, MA: Harvard University Press; Archard, David. 2008. “Informed Consent: Autonomy and Self‐Ownership.” *Journal of Applied Philosophy* 25(1): 19–34. Conversely, ‘the right to bodily integrity’ has been used to refer to several other kinds of right over the body and is often used without its precise content being specified. See, for discussion, Viens, A. M. 2020. “The Right to Bodily Integrity.” In *Cambridge Handbook of New Human Rights*, edited by Andreas von Arnauld, Kerstin von der Decken, and Mart Susi, 363–77. Cambridge: Cambridge University Press; Dekkers, W., C. Hoffer, and J. P. Wils. 2005. “Bodily Integrity and Male and Female Circumcision.” *Medicine, Health Care and Philosophy* 8(2): 179–91.

24 For examples of explicit invocations of the right in relation to organ harvesting, see Wilkinson, S., and E. Garrard. 1996. “Bodily Integrity and the Sale of Human Organs.” *Journal of Medical Ethics* 22(6): 334–39, p. 338 (‘[o]ne way of explaining the moral significance of organ removal is by appealing to the notion of bodily integrity’); and Wilkinson, T. M. 2011. *Ethics and the Acquisition of Organs*. Oxford: Oxford University Press, p. 16 (‘[t]he right to bodily integrity … is almost entirely uncontroversial and often considered of great weight’).

25 See, for example, Thomson op. cit.; Ripstein, Arthur. 2006. “Beyond the Harm Principle.” *Philosophy and Public Affairs* 34(3): 215–45; Archard op. cit.

26 For the classic discussion of this requirement, see Bovens, Luc. 2009. “The Ethics of Nudge.” In *Preference Change: Approaches from Philosophy*, edited by T. Grüne‐Yanoff and S. Hansson, 207–19. Dordrecht: Springer.

27 See, for discussion of this requirement, Thaler and Sunstein 2008 op. cit., p. 6; Saghai, Y. 2013. “Salvaging the Concept of Nudge.” *Journal of Medical Ethics* 39(8): 487–93.

28 For discussion of this requirement, see Engelen, B. 2019. “Nudging and Rationality: What Is There to Worry?” *Rationality and Society* 31(2): 204–32; Schmidt, A. T. 2019. “Getting Real on Rationality – Behavioral Science, Nudging, and Public Policy.” *Ethics* 129(4): 511–43.

29 Schmidt op. cit. offers an argument for the view that paradigmatic nudges, such as *Cafeteria Nudge*, need harness no irrationality, and there is nothing in his argument to prevent it carrying over to *Cafeteria Spray*.

